# Transient Receptor Potential Channels in Physiology and Pathophysiology: Special Issue

**DOI:** 10.3390/ijms26104960

**Published:** 2025-05-21

**Authors:** Mohammad Shahidullah, Paul J. Donaldson

**Affiliations:** 1 Department of Physiology, College of Medicine, The University of Arizona, Tucson, AZ 85724, USA; 2 Department of Physiology, School of Medical Sciences, Aotearoa New Zealand National Eye Centre, University of Auckland, 85 Park Road, Auckland 1023, New Zealand; p.donaldson@auckland.ac.nz

Since the discovery of the first family member by Cosens and Mannings over 50 years ago [1], Transient receptor potential (TRP) channels have emerged as an extremely important and unique family of membrane proteins. There are currently some 29 different family members that are grouped into 7 subfamilies based on amino acid sequence homology: TRPA (Ankyrin), TRPC (Canonical), TRPM (Melastatin), TRPP (Polycystin),TRPML (Mucolipin), and TRPV (Vanilloid) while TRPN (No-mechano-potential) is expressed predominantly in invertebrates like fruit flies, worms, as well as some lower vertebrates like zebrafish and has not been reported in mammals. Since their first discovery there has been an impressive expansion not only in the numbers of proteins identified, but also the mapping of their wide tissue distribution patterns and the characterization of the numerous vital functions performed by TRP channels. TRP channels are predominantly located on plasma membranes, but some, namely TRPM2, TRPM8, TRPV1, TRPP1, TRPA1, and TRPV4 are also reported to be located on intracellular membranes [2].

At the functional level, TRP channels mediate a diverse range of processes such as ion transport and homeostasis, fluid secretion, inflammation, pressure and thermoregulation, vision, taste, sense, and smell. Unlike many other receptor proteins, TRPs are ‘polymodal’ meaning they can be activated by multiple types of stimuli, including temperature, chemical ligands, pressure, osmotic stress and voltage. This polymodality coupled with the large number of family members gives rise to tremendous functional diversity. The channels are permeable to a wide range of cations, predominantly Ca^2+^, Mg^2+^, Na^+^, K^+^ and this differential selectivity or permeability to different cations further contributes to the functional diversity of the TRP channel family. For example, TRPV5 and TRPV6 are exquisitely Ca^2+^-selective [3], TRPM4 and TRPM5 mainly carry Na^+^ ions with negligible permeability to Ca^2+^ [4,5], while TRPM7 has the highest permeability to Zn^2+^ and least permeability to Ca^2+^ [6]. Furthermore, some TRP channel proteins, such as TRPM7, exhibit dual functionality, acting as both a channel and an enzyme (chanzyme), which adds yet another level of functional diversity [7]. Hence, given the sheer number of physiologically important functions this family of proteins perform, dysfunction of TRP channels has been linked to numerous diseases and have either been identified or promoted as the potential targets for pharmacological intervention. In this current Special Issue of the International Journal of Medical Sciences, the assembled research and review articles illustrate not only the functional diversity of TRP channel family, but also how this diversity contributes to the normal and patho-physiology in a wide variety of tissue types with distinctly different cellular processes.

The eye, for example, is comprised of a variety of different tissue types and these tissues utilize different TRP channel iosforms to regulate specific cellular processes to control ocular function and therefore vision quality. Frutos-Rincon and co-workers showed a role for TRPA1 in sensing ocular surface temperature and thermally evoked reflex blinking [8]. Interestingly, their findings indicated that absence of TRPA1 does not eliminate reflex blinking, but its presence equipped the corneal cold thermoreceptors to codify the intensity of temperature change so that the blink reflexes become proportional to the temperature. The implication of this finding is that TRPA1 or the temperature stimulus might not be the only entity controlling blinking. Indeed, in earlier work TRPM8 was identified as a peripheral osmosensor responsible for the regulation of normal eye-blinking in mice [9]. Further, TRPM8 is regarded as a cold receptor [10] and cold sensitivity of corneal primary sensory neurons has been documented [11]. Future work to determine the specific and proportional roles of TRP channels would be interesting.

Still at the front of the eye Lucius and co-authors investigated the causes of dry eye disease (DED). DED is a multifactorial condition but tear hyperosmolarity is a common feature and osmoprotective agents are frequently used in the management of DED [12]. In their article they described the mechanism of action of the endogenous osmoprotectant, L-carnitine, through inhibition of TRPV1 activation [13]. Interestingly L-carnitine was shown to prevent TRPV1 activation induced increase in intracellular calcium, whole cell current and cell volume shrinkage. The finding is significant in that TRPV1 is implicated in both the initiation and progression of DED [14] and L-carnitine appears to selectively inhibit TRPV1. It would be interesting to find the mechanism of other osmoprotectants such as erythritol, taurine and trehalose.

In the eye the aqueous humor is secreted by the ciliary epithelium which is composed of a bilayer of pigmented and non-pigmented cells that are connected at their apical-apical interface by gap junction channels [15,16]. Using primary cultured porcine nonpigmented ciliary epithelium Shahidullah and Delamere [17] showed that TRPV4 agonist, mechanical stretch or hypoosmotic swelling stretch opens hemichannels as determined by increased ATP release or propidium iodide uptake. The responses were abolished either by using selective TRPV4 antagonists or inhibitory connexin mimetic peptide Gap-27. Earlier and current findings suggest TRPV4 opens HC in a physiologically useful and controlled manner that prevents intracellular calcium concentration reaching a detrimental level [18,19,20] Future works need to be directed towards finding the mechanism of this interaction between TRPV4 and connexin HC, i.e., whether they are physically interacted due to proximity or a functional interaction through intermediary signaling molecules.

In the lens it has been shown that water/ion homeostasis or hydrostatic pressure is maintained by a dual feedback mechanism constituted by TRPV1 and TRPV4 [21,22,23]. Nakazawa and his coworkers [24] have now shown that alterations in the tension applied to the lens via the zonules that attach the lens to the ciliary muscle, osmotic challenge to the lens and application of pharmacological activators can modulate the trafficking of TRPV1 and TRPV4 in the peripheral lens fibers. Their findings suggest that these challenges reciprocally changed the distribution of two mechanosensitive TRP channels, i.e., when TRPV1 moved to the membrane, TRPV4 moved to the cytoplasm and vice versa, supporting the finding that lens water transport is dynamically regulated to maintain the optical properties of the lens [25].

Finally, at the back of the eye TRP channels appear to be involved in sensing of light. Light-induced activation of the native Drosophila TRP/TRPL channels is an extremely fast event. An essential step in this event is the production of diacylglycerol (DAG) by phospholipase C (PLC)-mediated hydrolysis of membrane phospholipid PIP2. To explain the fast light-induced TRPL current, Dr Baruch Minke’s group conducted an interesting study in which standard and optically activated DAG analogs were added to the cytosol using the patch-clamp pipette of TRPL-expressed HRK cells [26]. Their data showed that the activation of exogenously expressed TRPL channels in HEK cell was much slower in response to either the normal DAG analog, 1-oleoyl-2-acetyl-sn-glycerol (OAG), or to the optically activated DAG analog, OptoDArG. They concluded that DAG alone was not sufficient for TRPL channel activation under physiological conditions. Although genetically expressed protein usually localizes correctly, factors like experimental conditions or absence of proper signal sequences to direct the encoded protein to its correct location can lead to mislocalization. The consequences are that the encoded protein’s expression pattern, dynamics and subcellular localization in relation to its interacting partners in the signaling cascade might not mimic the in vivo pattern. It would be interesting to verify the encoded protein’s location in relation to the signaling partners using appropriate techniques like immunofluorescence microscopy or gene tagging strategies [27].

As well as being involved in a variety of ocular tissues that TRP channels have also been shown to be involved in the ear. Earlier studies had shown that TRPC1, TRPC3, TRPC5 and TRPC6 quadruple KO mice have larger hearing deficit than TRPC3 and TRPC6 double KO mice [28]. However, single KOs had no defect. In this special issue Englisch and coworkers [29] used immunohistochemistry to show for the first time the expression/localization of TRPC6, TRPC5 and TRPC3 in key structures of human cochlea supporting critical roles of TRPC channels in human cochlear health and disease. One interesting aspect of their work is the description of a novel technique to overcome the difficulty in isolating intact cochlea from human skull using computed tomography (CT). While their findings are interesting, they used prolonged formalin preserved tissues of old donors of 84.2 years. This deficiency renders the findings less useful in explaining true pathophysiological implications. Future study would be necessary using tissues from young individuals or young age-matched monkey with normal hearing to reflect in vivo condition.

Aspiration pneumonias is a prevalent and important problem in aged, post-stroke patients and individuals with neurodegenerative diseases. It appears that multiple TRP channels, including TRPV1, TRPA1 and TRPM8 are involved in the neurophysiology of swallowing. In this issue Tomsen and coworkers [30] investigated the effects of multiple TRP channel agonists on biomechanics of swallowing and on the functionality of afferent (sensory) and efferent (motor) pathways of swallowing in patients with oropharyngeal dysphagia (OD). Their results suggested that, in contrast to thickening products, TRPV1 and TRPV1/A1 agonists could form the basis of pharmacological strategies to treat compromised neurophysiology and biomechanical swallow function in patients with chronic OD. Comprehensive future studies defining the role of individual TRP channel and long-term treatment to determine any receptor desensitization and unwanted effects would be interesting.

TRPC and TRPM channels have been implicated in the accumulation of excessive amounts of free zinc along with calcium in postsynaptic neurons in several brain diseases, including ischemic stroke, epilepsy, and traumatic brain injury [31]. Excessive free zinc in the cytoplasm is detrimental to cells by promoting oxidative stress, apoptosis and cell death. Based on a brief review of this field Hong et al. suggested that the inhibition of TRPC and TRPM channels by antagonists may be neuroprotective in brain disease. Overwhelming evidence suggested that connexin hemichannels in the glia play an important role in many neurogenerative brain diseases [32]. Recent evidence suggests activation of TRPV4, TRPV1 and connexin 43 and pannexin 1 hemichannels in epilepsy including neuroinflammation and oxidative stress [33]. Calcium and zinc overload, oxidative stress, cellular mediator release such as ATP, ADP, and glutamate appear to be important common features of many traumatic or neurogenerative brain diseases. Thus, to have clearer ideas on the roles of these channels future studies should be directed to reveal interaction of TRP channels with Hemichannels in brain diseases. Interaction of TRPV4 with connexin has been reported [17,18,19].

The research article by Demartini and coworkers presented data showing TRPA1 antagonism reverts experimentally induced hyperalgesia in nitroglycerin-induced acute and chronic rat models of migraine and prevents multiple changes in inflammatory pathways by modulating glial activation [34]. Other TRP channels were reported to be involved in nociception. TRPV1 and TRPM3 were previously shown to play roles in migraine pathophysiology [35,36,37]. One future direction could be to find the proportional and specific roles of different TRP channels expressed in sensory neurons, namely TRPA1, TRPV1, TRPV4 and TRPM3.

Mack and his coworkers used transgenic rats containing Gag & Pol deleted HIV-1 viral genome (HIV-1 Tg rats) that mimic HIV-1 patients on combination anti-retroviral therapy (cART) and showed EtOH and HIV-1 protein reduced TRPM7 expression in rat brain microvascular endothelial cells (BMVEC) [38]. Their data also show EtOH and HIV-1 protein similarly reduced TRPM7 expression in human BMVEC. In patients on cART viral replication is inhibited. Gag gene codes for structural proteins for the virus. Pol gene codes for reverse transcriptase, protease, and integrase required for viral replication. Thus, HIV-1Tg rats persistently express 7 of the 9 HIV-1 genes but viral replication is inhibited mimicking patients on cART. Since brain microvascular endothelium constitutes BBB, they suggested that TRPM7 could be involved in the mechanisms underlying blood brain barrier (BBB) alcohol-induced damage in HIV-1 patients on cART. Their data has some added significance in that they used human BMVEC for both TRPM7 expression and BBB permeability studies in response to EtOH, HIV-1 protein and TRPM7 antagonist. Future studies could extend to using conditional Trpm7 knockout mice and cell lines as reported for enamel epithelium [39].

In their research article Lowin and colleagues showed that non-psychotropic, minor cannabinoid, cannabigerol (CBG) had broad anti-inflammatory effects in isolated RASF (rheumatoid arthritis synovial fibroblasts), PBMC (peripheral blood mononuclear cells) and PBMC/RASF co-cultures [40]. Although CBG interacts with several members of the transient potential receptor (TRP) family, TRPA1 is the main target with the highest affinity and potency. Based on the anti-apoptotic and nociceptive role of TRPA1, the authors suggested that CBG might be used as an add on therapy for rheumatoid arthritis. Rheumatoid arthritis is a complex and multifactorial discase [41,42] and progress in its prevention and treatment is highly desirable. However, CBG influences multiple biological targets. It should be interesting to see the short and long term CBG effects on animal models of Rheumatoid arthritis.

Vestuto and coworkers reviewed the role of TRP channels in ER stress [43]. ER is an extremely important subcellular organelle with myriads of functions in the maintenance of cellular homeostasis, intra and intercellular signaling, protein synthesis and folding, calcium storage and many more. TRP channels are no exception, they play such vital roles that life’s existence and interaction with surroundings are mostly dependent on these channels. Thus, this is an excellent attempt to compile and summarize the roles of TRP channels on ER stress. It is of particular interest to note that factors of ER stress are also numerous, including but not limited to nutrient deprivation, DNA damage, calcium depletion, oxidative stress, hypoxia and pH variations. The review made it clear that this is an inadequately explored area and further research should be directed towards finding detailed pathophysiology of ER stress involving TRP channels.

The last review article in the special issue by Englisch and coworkers was a compilation of current knowledge on the pathophysiological roles of TRPC3 and TRPC6 channels on renal tubular system [44]. While the physiological functions of TRPC6 still need to be convincingly demonstrated, TRRPC3 appears to perform numerous functions in the kidney, including regulation of glomerular filtration, intracellular Ca^2+^ reabsorption in the proximal tubule, acting as osmosensor and regulating water transport and Ca^2+^ reabsorption in the collecting ducts (CD) [45]. Since renal tissues expresses multiple TRP channels including TRPV1, TRPV4, TRPC3, TRPC5, TRPC6 [45,46,47,48], several TRPM isoforms [49], careful investigations using tissue-specific TRPC3 deleted animal models would be highly valuable in order to define the contribution of TRPC3 in renal functions.

In conclusion, the breath of topics covered in this special issue illustrates the functional diversity of TRP channels and hence their potential as therapeutic targets to treat a wide range of diseases across the full spectrum of neurodegenerative, metabolic, cardiovascular, inflammatory/immune and congenital disorders. We expect to see sprouting growth and discoveries on TRP channels in the next decade. However, the field faces some formidable challenges due not only to the large number of mammalians TRP channels (at least 28 have been identified to date) but also because of their overlapping and redundant functions which would make TRP-specific drugs prone to off target adverse side effects. Systemic adverse effects could be minimized by developing targeted, site-specific therapies. Additionally, research should be directed towards appropriate target engagement and safety biomarkers to define clinically useful doses and therapeutic index.
